# *YWHAZ* amplification/overexpression defines aggressive bladder cancer and contributes to chemo‐/radio‐resistance by suppressing caspase‐mediated apoptosis

**DOI:** 10.1002/path.5274

**Published:** 2019-04-29

**Authors:** Chia‐Cheng Yu, Chien‐Feng Li, I‐Hsuan Chen, Ming‐Tsung Lai, Zi‐Jun Lin, Praveen K Korla, Chee‐Yin Chai, Grace Ko, Chih‐Mei Chen, Tritium Hwang, Shan‐Chih Lee, Jim J‐C Sheu

**Affiliations:** ^1^ Department of Surgery Kaohsiung Veterans General Hospital Kaohsiung Taiwan; ^2^ Department of Pharmacy College of Pharmacy and Health Care, Tajen University Pingtung County Taiwan; ^3^ Department of Surgery Tri‐Service General Hospital, National Defense Medical Center Taipei Taiwan; ^4^ School of Medicine National Yang‐Ming University Taipei Taiwan; ^5^ Department of Pathology Chi‐Mei Medical Center Tainan Taiwan; ^6^ National Institute of Cancer Research National Health Research Institutes Miaoli Taiwan; ^7^ Department of Biotechnology Southern Taiwan University of Science and Technology Tainan Taiwan; ^8^ Department of Pathology Taichung Hospital, Ministry of Health and Welfare Taichung Taiwan; ^9^ Human Genetic Center China Medical University Hospital Taichung Taiwan; ^10^ Department of Medical Imaging and Radiological Sciences Chung Shan Medical University Taichung Taiwan; ^11^ Institute of Biomedical Sciences National Sun Yatsen University Kaohsiung Taiwan; ^12^ Department of Medicine Kaohsiung Medical University Kaohsiung Taiwan; ^13^ Department of Medical Imaging Chung Shan Medical University Hospital Taichung Taiwan; ^14^ School of Chinese Medicine China Medical University Taichung Taiwan; ^15^ Department of Health and Nutrition Biotechnology Asia University Taichung Taiwan

**Keywords:** urothelial carcinomas of the urinary bladder (UCUB), YWHAZ (14‐3‐3 ζ/δ), caspase, apoptosis, chemo‐resistance, radio‐resistance

## Abstract

The objective of this study was to characterize the oncogenic actions of a recently identified cancer‐associated gene *YWHAZ* (also named as 14‐3‐3 ζ/δ) in urothelial carcinomas of the urinary bladder (UCUB). A genome‐wide study revealed *YWHAZ* to be involved in the amplicon at 8q22.3, and its genetic amplification was detected predominantly in muscle‐invasive bladder cancer (MIBC). Immunohistochemical staining confirmed the association of YWHAZ overexpression with higher tumor stages, lymph node/vascular invasion, and mitotic activity. Univariate and multivariate analyses further indicated the prognostic potential of YWHAZ for more aggressive cancer types. Both gene set enrichment analysis and STRING network studies suggested involvement of YWHAZ in regulating caspase‐mediated apoptosis. Ectopic expression of YWHAZ in bladder cells with low endogenous YWHAZ levels boosted cell resistance to doxorubicin and cisplatin, as well as to ionizing radiation. Conversely, YWHAZ‐knockdown using specific shRNA in cells with high endogenous YWHAZ levels diminished survival activity, suppressing cell growth and increasing cell death. Our findings confirm the essential role played by YWHAZ in sustaining cell proliferation during chemo/radiotherapy. Treatments based on anti‐YWHAZ strategies may thus be beneficial for UCUB patients overexpressing YWHAZ. © 2019 The Authors. *The Journal of Pathology* published by John Wiley & Sons Ltd on behalf of Pathological Society of Great Britain and Ireland.

## Introduction

Bladder cancer is the most common tumor of the urinary collecting system, and transitional cell carcinomas (urothelial carcinoma of the urinary bladder; UCUB) account for more than 90% of malignant tumors of the bladder 1. Risk factors causally related to the development of bladder cancer include cigarette smoking, occupational exposure to chemicals, and certain viral infections. Approximately 80% of all new cases of UCUB are classified as nonmuscle invasive (NMIBC; stages Ta/T1), also called superficial bladder cancer, while the remaining 20% of cases are muscle invasive (MIBC; stages T2–4) 1. Currently, the prognoses and treatments for these two types of UCUB differ markedly due to their distinct clinical features and outcomes. Recent studies using integrative cancer genomics approaches have advanced our understanding of the genomic landscapes of these two types of UCUB 2, 3, 4. However, their respective origins remain controversial 5, 6, 7, 8. Thus, molecules involved in the interplay between these two UCUB types are interesting targets to explore.

YWHAZ is a member of the 14‐3‐3 protein family and it functions as a central hub protein for many signal transduction pathways 9. Previous studies have shown that proteins in the 14‐3‐3 family are highly conserved and ubiquitously expressed in all eukaryotic organisms 9, 10. Although not functioning as enzymes, 14‐3‐3 proteins can form homo/heterodimers and bind to phosphorylated serine/threonine motifs on their target proteins, thereby altering the activity of their targets through post‐translational regulation 11. Upregulation of 14‐3‐3 isoforms was recently implicated in a variety of human cancers; however, understanding their specific clinical and biological significance awaits further investigation.

In recent years, YWHAZ has gained attention because its elevated expression associates with a variety of cancers, which indicates it may function as an oncoprotein 12, 13, 14, 15, 16, 17, 18, 19, 20. Clinicopathologic studies further support the relevance of YWHAZ in cancer malignancy and lymph node metastasis 18, 21. In particular, YWHAZ interacts with many apoptotic proteins, including NOXA, BAD, BAX, Raf kinases and caspase‐2, suggesting it plays a critical role in regulating apoptosis and allowing cellular adaptation to environmental stresses 22, 23, 24, 25. In addition, YWHAZ has been found to act as a critical regulator that switches TGF‐β's function from tumor suppressor to metastasis promoter through contextual changes of Smad's partner from p53 to Gli2 26. This evidence supports the idea that activities associated with aberrant YWHAZ expression contribute to its oncogenic function in UCUB by enhancing survival activity.

In the present study, we identified YWHAZ as a possible cancer driver that its amplification/overexpression associates with UCUB progression and poor clinical outcomes. Downstream network analyses and *in vitro* functional studies confirmed that UCUBs overexpressing YWHAZ gain the capacity to tolerate environmental stresses, including chemo/radiotherapy. Therefore, targeting YWHAZ may be an effective strategy for treating UCUBs exhibiting drug and/or radiation resistance.

## Materials and methods

### Cell lines and reagents

The BFTC905, HT1376, T24, 5637, TSGH8301, and RT4 human bladder cancer cell lines were purchased from the Bioresource Collection and Research Center (BCRC), Taiwan. These cells were cultured in Dulbecco's modified Eagle's medium supplemented with 10% fetal bovine serum and 1% penicillin/streptomycin mixture (Gibco/Thermo Fisher Scientific, Waltham, MA, USA) under a humidified atmosphere containing 5% CO_2_ at 37 °C. Cisplatin and doxorubicin were purchased from Merck (Darmstadt, Germany). For Western blotting, anti‐PARP‐1 (HPA045168) Ab was purchased from Sigma–Aldrich Corp. (St. Louis, MO, USA); anti‐YWHAZ (NBP1‐61188), anti‐BAk1 (NBP2‐67460), and anti‐BAX (NBP1‐28566) Abs were from Novus Biologicals LLC. (Littleton, CO, USA); anti‐CASP3 (ab13585), and anti‐CASP10 (ab177475) Abs were from Abcam (Cambridge, UK); and anti‐CASP7 (MAB6243) Abs were from Abnova Corp. (Taipei, Taiwan).

### Human samples and tissue microarray preparation

Ten samples of normal urothelium and 60 UCUB samples (20 at stage Ta‐T1, 10 at stage T2, 17 at stage T3, and 13 at stage T4) were freshly collected from clinics and/or BioBank at the Kaohsiung Veterans General Hospital (KVGH) and Chi‐Mei Medical Center (CMMC) with the informed consent of each participant. The cancer tissues were initially stored in liquid nitrogen and then verified to contain >85% tumor cells by staining the frozen sections with hematoxylin. For tissue microarray preparation, 295 formalin‐fixed, paraffin‐embedded bladder cancer samples and 20 urothelium samples (three representative cores from each sample) were arranged onto tissue microarrays. The histological grading of UCUB was done according to the WHO classification. The tumor stages were determined based on American Joint Committee on Cancer (AJCC) TNM system in which primary tumor status and nodal metastasis were designated as T and N statuses, respectively. This study was reviewed and approved by the institutional review boards of KVGH and CMMC (VGHKS16‐CT11‐17 and 10302‐015, respectively).

### DNA isolation for copy number alteration analysis

Genomic DNA was purified from frozen tissue samples using a DNeasy kit (Qiagen, Chatsworth, CA, USA) and subjected to genotyping using 250 K SNP arrays (Affymetrix, Santa Clara, CA, USA) according to the procedures provided by the manufacturers. Additional details of the method can be found in our earlier study 27. DNA samples from two cell lines, BFTC905 and TSGH8301, were similarly studied. The dChip program was used to normalize chip data using a model‐based (PM/MM) method, and copy number alterations in each sample were analyzed using the Hidden Markov Model with the assumption of diploidy for normal samples. Based on our earlier study 27, an amplified region with a cutoff of >2.6 copies in more than three consecutive SNPs was defined as an amplicon or genetic gain.

### Dual‐color fluorescence *in situ* hybridization (FISH)

To confirm YWHAZ genetic gain/amplification in UCUBs, bacterial artificial chromosome clones RP11‐102K7 (red signal for 8q22.2 amplicon) and RP11‐327O12 (green signal for control) were purchased from Thermo/Invitrogen (Carlsbad, CA, USA). Based on our SNP array data, the RP11‐327O12 clone covers a region on chromosome 8 with no obvious copy number change in the UCUBs tested. The method for dual‐color FISH on tissue microarrays was described previously 28. Approximately 100 tumor cells per sample were examined under a fluorescence microscope (IX83, Olympus Corp., Shinjuku, Japan) and genetic amplification/gain was defined as a red‐to‐green signal ratio exceeding 2.5.

### Immunohistochemistry

Mouse anti‐YWHAZ monoclonal antibodies (NBP1‐61188) were utilized for IHC studies using standard protocols with an Ab dilution of 1:100. Chromogen color was developed using an EnVision+System peroxidase kit (DAKO, Carpinteria, CA, USA) and the immunointensity of YWHAZ staining was scored independently by two pathologists and labeled as negative (0), weakly positive (+1), moderately positive (+2), strongly positive (+3), or intensely positive (+4). In cases where there was not consensus, a third investigator was invited to score the sample, and the final score was determined by the majority scores.

### Data mining and functional interaction networks

The SNP array data from Dana‐Farber Cancer Institute (GSE39282) 29 were collected from the GEO data bank (https://www.ncbi.nlm.nih.gov/gds/) to validate our SNP array data. To know the biological relevance of genetic statuses, mRNA expression and protein levels of *YWHAZ* in UCUBs, data of the TCGA cohort 29, 30 were obtained from The Cancer Genome Atlas (TCGA) database via cBioportal (www.cbioportal.org). To map the key downstream pathways, normalized gene expression data (*n* = 404) were collected from the TCGA database, and gene set enrichment analysis (GSEA) was performed using GSEA Java (version 2.2.3) from Broad Institute 31, using which the analyses were based on the rank of mRNA expression levels in correlation with the *YWHAZ* expression. Genes up‐ or downregulated along with *YWHAZ* amplification/overexpression, with *P* values <1.00E−05 and false discovery rates (*q* values) < 1.00E−03, were also filtered out. The functional interactome among the up‐/downregulated genes were analyzed using the STRING protein–protein interaction database (http://string‐db.org/).

### Cell‐based functional studies

YWHAZ cDNA was amplified using nested PCR and then cloned into the vector pcDNA6B/V5 (Clontech Lab., Mountain View, CA, USA). The resultant YWHAZ expression vector was transfected into RT4 cells using Lipofectamine 3000 (Thermo/Invitrogen), and the transfected cells were further enriched by incubation in the presence of 7 μg/ml blasticidin (Thermo/Invitrogen). Cells transfected with empty vector served as controls. For gene knockdown, specific shRNAs against *YWHAZ* and the scrambled control were purchased from the miRNA core at Academic Sinica, Taiwan. Growth of transfectants expressing different constructs was monitored daily using Alamar Blue assays (Thermo Fisher Scientific, Hampton, NH, USA). To assess cell viability, cells were stained with annexin V and propidium iodide (BD Biosciences, Franklin Lakes, NJ, USA) 48 h after drug or radiation treatments. To assay radiosensitivity, cells were exposed to gamma radiation generated using a Clinac‐6Ex electron linear accelerator (Varian Medical Systems, Inc., Palo Alto, CA, USA) with an electron beam energy of 6 MeV. Data were expressed as means ± SD from five replicates in each experimental group.

### Quantitative reverse transcription polymerase chain reaction

RNA samples were collected from cells with different treatments using an RNA isolation kit (Qiagen), and the cDNA were prepared by using M‐MuLV reverse transcriptase enzyme according to the procedure provided by the manufacture (NEB Inc., Ipswich, MA, USA). Changes in the mRNA levels of *YWHAZ* and the downstream effectors were detected by SYBR Green‐based real‐time qPCR (Thermo Fisher Scientific) with the primers as listed in supplementary material, Table S1. Levels of *GAPDH* served as the internal control for qPCR data normalization. Data were expressed as means ± SD from triplicates in each experimental group.

### Statistical analyses

SPSS V.14.0 software (SPSS Inc., Chicago, IL, USA) and GraphPad Prism (GraphPad Software Inc., San Diego, CA, USA) were utilized for statistical analyses. Statistical differences between two groups were evaluated using a *t*‐test. One‐way ANOVA was used to compare differences among multiple groups. Survival curves were sketched by the Kaplan–Meier method, and a log‐rank test was performed to assess prognostic significances. Parameters with univariate *P* values less than 0.05 were enrolled in multivariate tests conducted using a Cox proportional hazards model. Values of *p* < 0.05 were considered statistically significant.

## Results

### *YWHAZ* genetic gain/amplification is more frequent in advanced muscle‐invasive bladder cancer

**Figure 1:**
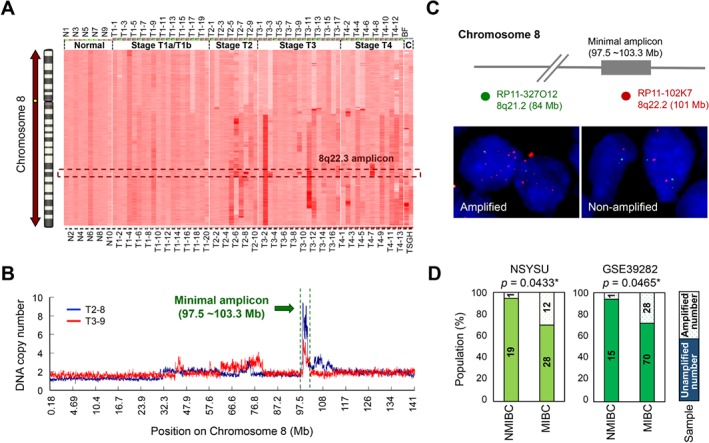
*YWHAZ* amplification/genetic gain is more frequently detected in MIBC. (A) Genetic alterations in 60 UCUB samples (20 at stage Ta‐T1, 10 at stage T2, 17 at stage T3, and 13 at stage T4) and 2 cell lines were determined using 250K SNP arrays. The SNP values on chromosome 8 were normalized to the average data from 10 samples of normal urothelium and are shown in a heatmap alone with genetic loci on the chromosome. (B) The minimal 8q22.3 amplicon was mapped to 97.5–103.3 Mb by overlapping amplified regions (with copy numbers >2.5) in UCUBs. (C) Dual‐color FISH was performed on UCUB tissue blocks. Red signals (RP11‐102K7) indicate the 8q22.3 amplicon region, while green signals (RP11‐327O12) indicate the control region on chromosome 8, with genetic copy numbers near 2.0. (D) Statistical analysis of 8q22.3 amplicon occurrence between NMIBC (stages Ta to T1) and MIBC (stages T2–T4) using a proportional *t*‐test. Two study cohorts were analyzed: one from our group (NSYSU; *n* = 60) and another from Dana–Farber Cancer Institute (GSE39282; *n* = 114). *p < 0.05, **p < 0.01, ***p < 0.001.

A total of 72 urothelium samples, including 10 samples of normal bladder tissue; 20 UCUBs at stage Ta–T1, 10 at stage T2, 17 at stage T3, and 13 at stage T4; and 2 bladder cancer cell lines, were collected for genomic alteration analysis using a genome‐wide approach. Among the regions exhibiting genetic aberrations, we discovered a unique amplicon at chromosome 8q22.3, which was frequently detected in cancers at more advanced stages (T2–T4, muscle‐invasive bladder cancer [MIBC]) but rarely found in early stage cancers (T0/T1, nonmuscle‐invasive bladder cancer [NMIBC]) (Figure 1A,D, *p* = 0.043). Through amplicon mapping, we identified the smallest amplified region to be 97.5–103.3 Mb on chromosome 8 containing 28 annotated genes (Figure 1B; see supplementary material, Table S2). In addition to some RNA‐processing genes, *YWHAZ* was revealed to be the most cancer‐related gene in the 8q22.3 amplicon. Dual‐color FISH confirmed the existence of *YWHAZ* gain or amplification in UCUBs (Figure 1C), especially advanced‐stage cancers (T2–T4) (*p* < 0.001) with lymph node invasion (*p* = 0.027) and higher mitotic activity (HPF ≥ 10) (*p* = 0.003) (Table 1). SNP array data of the study cohort from Dana‐Faber Cancer Institute (GSE39282) containing 16 NMIBCs and 98 MIBCs were utilized to validate our data, and it confirmed a tendency toward *YWHAZ* genetic gain/amplification in MIBCs (Figure 1D, *p* = 0.047). Our data suggest an association between *YWHAZ* amplification/overexpression and more advanced UCUBs.

**Table 1:** Correlation between *YWHAZ* amplification/protein overexpression and various clinicopathological factors

		14‐3‐3 ζ/δ (*n* = 295)			*YWHAZ* gene (*n* = 289)		
Parameters	Category	No. of case	Low (0 to ∼2+)	High (3+ to ∼4+)	*P* value	No. of cases	No amp.	Amp.	*P* value
Gender	Male	216	161	55	0.282	213	122	91	0.416
	Female	79	64	15	76	48	28
Age (years)	<60 years	81	66	15	0.222	80	48	32	0.894
	≧60 years	214	159	55	209	122	87
Primary tumor (T)	Ta	84	74	10	<0.001***	83	59	24	<0.001***
	T1	88	75	13	85	56	29
	T2–T4	123	76	47	121	55	66
Nodal status (N)	N0	266	210	56	0.002**	260	109	101	0.027*
	N1–N2	29	15	14	29	11	18
Histological grade	Low grade	56	48	8	0.080	55	38	17	0.095
	High grade	239	177	62	234	132	102
Vascular invasion	Absent	246	195	51	0.010*	240	144	96	0.426
	Present	49	30	19	49	26	23
Perineurial invasion	Absent	275	213	62	0.100	269	160	109	0.482
	Present	20	12	8	20	10	10
Tumor necrosis	Absent	191	148	43	0.567	186	112	74	0.535
	Present	101	77	27	103	58	45
Mitotic activity (10 HPF)	<10	139	115	24	0.019*	135	92	43	0.003**
	≥10	156	110	46	154	78	76

Amp, amplification. Statistically significant:

* *p* < 0.05

** *p* < 0.01

*** *p* < 0.001.

### YWHAZ overexpression defines poor clinical outcomes

**Figure 2:**
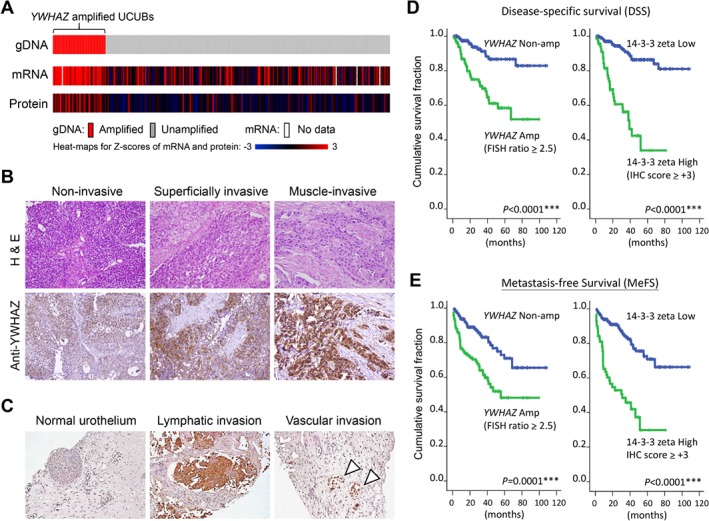
*YWHAZ* amplification/overexpression correlates with poor clinical outcome. (A) Data of copy number variations, mRNA expression (RNA‐seq) and protein expression (RPPA) in UCUBs from the TCGA group were collected using cBioportal (www.cbioportal.org) and aligned according to their genetic statuses. (B) Immunohistochemistry was performed with tissue microarrays containing UCUBs at different histopathological stages. Representative images of staining for cancers at noninvasive (Ta), superficially invasive (T1) and muscle‐invasive (T2–T4) statuses are shown together with the corresponding images of H&E staining. (C) Representative IHC images of bladder cancer cells undergoing lymphatic (middle) and vascular (right) invasion. Staining of normal urothelium (left) was used as the control. Kaplan–Meier survival analyses of UCUB patients were performed to compare median (D) DSS and (E) MeFS times based on *YWHAZ* genetic contents by dual‐color FISH and protein expression levels detected by IHC.

Based on the dataset from TCGA 30, samples with *YWHAZ* genetic gain/amplification tended to also express higher mRNA (by RNA seq) and protein (by RPPA; reverse phase protein arrays) levels in their cancer tissues (*p* = 0.013) (Figure 2A). This suggests YWHAZ could potentially serve as a cancer driver during UCUB development. The clinical impact of YWHAZ expression was assessed using IHC with bladder cancers at different clinical stages. Our data indicate a correlation between YWHAZ upregulation and tumor staging (Figure 2B). Using our criteria for IHC scoring (see Materials and methods), cancer samples were stratified into *Low* (with scores of 0 to +2) and *High* YWHAZ expression (scores of +3 or +4) groups. Consistent with the FISH results, cancers with high YWHAZ levels associated with muscle‐invasive cancer type (*p* < 0.001) and higher mitotic activity (HPF ≥ 10) (*p* = 0.019) (Table 1). Notably, cancers with high YWHAZ levels showed a higher tendency to invade the lymphatic and vascular systems (Table 1; *p* = 0.002 and *p* = 0.010, respectively). IHC staining also revealed highly YWHAZ‐positive cells with invasions into lymphatic and vascular systems (Figure 2C). Univariate and multivariate analyses revealed that both *YWHAZ* genetic gain/amplification and protein overexpression correlated with shorter disease‐specific survival (DSS) and metastasis‐free survival (MeFS) times (Table 2). Kaplan–Meier survival analyses confirmed patients with IHC scores of 0 to +2 had a better median DSS and MeFS times than those with scores of +3 to +4 (Figures 2D,E). These data suggest that YWHAZ overexpression is an indicator of aggressive phenotypes and poor prognosis for UCUB patients as other key clinical features such as tumor stage or mitotic activity.

**Table 2:** Univariate and multivariate log‐rank analyses for DSS and MeFS

			Univariate analysis				Multivariate analysis					
			DSS		MeFS		DSS			MeFS		
Parameters	Category	Case no.	Event	*P* value	Event	*P* value	HR	95% CI	*P* value	HR	95% CI	*P* value
Gender	Male	216	39	0.640	59	0.400						
	Female	79	11		16							
Age (years)	<60	81	10	0.218	19	0.498						
	≧60	214	40		56							
Primary tumor (T)	Ta	84	1	<0.001***	4	<0.001***	1	‐	<0.001***	1	‐	0.004**
	T1	88	9		23		3.00	1.35–6.71		6.22	1.74–22.27	
	T2–4	123	40		48		27.78	2.83–250.00		7.77	2.16–27.98	
Nodal status (N)	N0	266	41	0.002**	61	<0.001***	1	‐	0.702	1	‐	0.276
	N1–2	29	9		14		1.17	0.51–2.56		1.43	0.75–2.70	
Histological grade	Low	56	2	0.001**	5	<0.001***	1	‐	0.970	1	‐	0.572
	High	239	48		70		1.03	0.19–4.91		1.36	0.47–3.99	
Vascular invasion	Absent	246	36	0.002**	54	<0.001***	1	‐	0.438	1	‐	0.920
	Present	49	14		21		1.33	0.37–1.54		1.10	0.53–1.76	
Perineurial invasion	Absent	275	44	0.005**	65	<0.001***	1	‐	0.145	1	‐	0.133
	Present	20	6		10		2.02	0.79–5.17		1.76	0.84–2.02	
Tumor necrosis	Absent	191	30	0.336	48	0.728						
	Present	104	20		27							
Mitotic activity (HPF)	<10	139	12	<0.001***	22	<0.001***	1	‐	0.025*	1	‐	0.020*
	≥10	156	38		53		2.18	1.10–4.29		1.87	1.11–3.15	
YWHAZ protein	Low (0 to ∼2+)	225	20	<0.001***	39	<0.001***	1	‐	<0.001***	1	‐	<0.001***
	High (3+ to ∼4+)	70	30		36		4.31	2.33–7.97		3.28	2.01–5.37	
*YWHAZ* gene	No amp.	170	14	<0.001***	30	<0.001***	1	‐	0.005**	1	‐	<0.001***
	Amp.	119	36		45		5.54	1.78–9.12		3.47	1.27–8.11	

Amp, amplification. Statistically significant:

* *p* < 0.05

** *p* < 0.01

*** *p* < 0.001.

### YWHAZ overexpression downregulates signaling for endopeptidase‐mediated cell death

**Figure 3:**
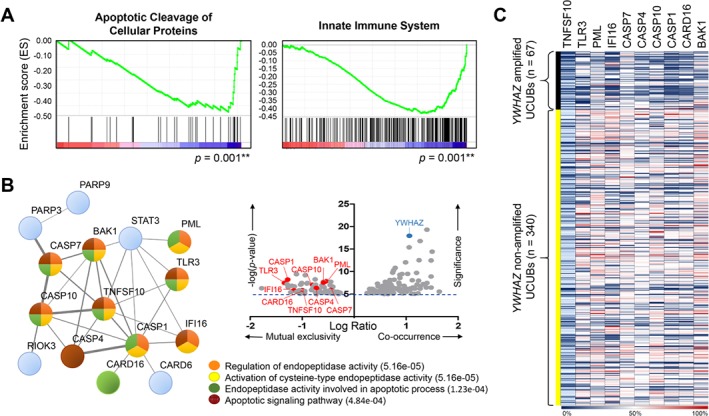
*YWHAZ* amplification/overexpression negatively regulates signaling pathways in caspase‐mediated apoptosis. (A) Gene set enrichment analysis was performed using mRNA expression data from the TCGA group. Two pathways, apoptotic cleavage of cellular proteins and innate immune system, were found associated with *YWHAZ* amplification/overexpression in UCUBs. (B) Scatter plot shows genes significantly (−log(*p* value) > 5.0) up‐ (log ratio > 0.3) or downregulated (log ratio < −0.3) along with *YWHAZ* amplification/overexpression (right upper panel). The functional protein interaction network of YWHAZ‐associated genes was analyzed using the STRING database (http://string‐db.org/) (left). Four major pathways were found to be involved in *YWHAZ* amplification/overexpression in UCUBs: regulation of endopeptidase activity, activation of cysteine‐type endopeptidase activity, endopeptidase activity involved in apoptotic process, and apoptotic signaling pathway with false discovery rates (*q* values) < 1.00 E‐03. (C) Gene expression heat map of 10 key genes involved in endopeptidase‐mediated cell death in UCUBs with or without *YWHAZ* amplification. Genetic and mRNA expression data were from the TCGA group.

An earlier study suggested the key role played by YWHAZ as a central hub involved in various signal transduction pathways, especially those contributing to apoptosis regulation 9. To assess the functional role of YWHAZ in bladder cancer, we performed GSEA using the TCGA cohort to define possible pathways regulated by *YWHAZ* amplification/overexpression in cancer tissues. Although the concurrently upregulated genes were very diverse in terms of their cellular functions (see supplementary material, Table S3), the major pathways regulated by the concurrently downregulated genes were apoptotic cleavage of cellular proteins (*p* = 0.001) and innate immune system (*p* = 0.001) (Figure 3A). Other related pathways with *P* values less than 0.01 are summarized in supplementary material, Figure S1 and Table S4. STRING network analysis of the mutually exclusive genes further confirmed the downregulation of pathways involved in endopeptidase‐mediated cell death in YWHAZ‐overexpressing UCUBs (Figure 3B). Expression levels of the 10 major genes involved in the endopeptidase‐mediated cell death (shown in Figure 3B) were found significantly lower in *YWHAZ* amplified UCUBs as compared to the levels in UCUBs without (all the *P* values were less than 0.001) (Figure 3C).

**Figure 4:**
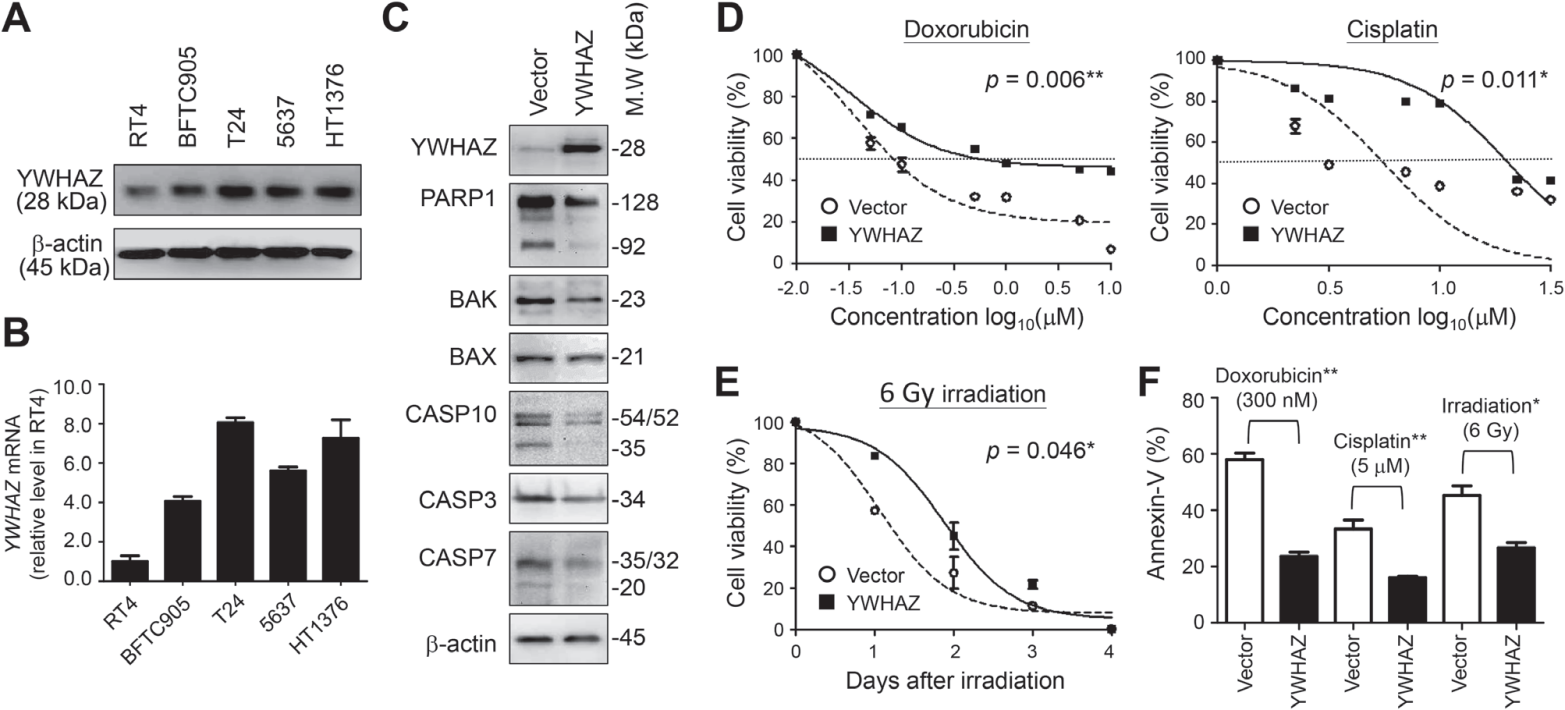
YWHAZ overexpression enhances survival activity to overcome environmental stresses induced by chemo−/radio‐therapy by downregulating caspase‐mediated apoptosis. (A) Western blotting was performed to determine endogenous YWHAZ levels in UCUB cell lines. Levels of β‐actin served as internal controls. (B) RT‐qPCR was performed to determine the relative expression levels of *YWHAZ* in UCUB cell lines using RT4 cells as the control. (C) Western blotting was performed to determine levels of the indicated effectors involved in caspase‐mediated apoptosis in RT4 cells 48 h after YWHAZ gene transfection. (D) RT4 cells overexpressing YWHAZ were treated with the indicated concentrations of doxorubicin and cisplatin. Cell viability was measured using Alamar Blue assays 4 days after drug treatments. (E) RT4 cells overexpressing YWHAZ were treated with 6 Gy irradiation, after which cell viability was measured every 24 h for 4 days. (F) Incidence rate of preapoptosis among treated cells 48 h after chemo−/radio‐therapy was determined by annexin‐V staining. Cells treated with empty vector served as controls in panels C to F. Data are expressed as means ± SD from five replicates in each experimental group. Paired *t*‐tests were performed to evaluate differences between vector‐ and YWHAZ‐treated cells. *p < 0.05, **p < 0.01, ***p < 0.001.

The above findings suggest bladder cancer cells expressing higher levels of YWHAZ are less susceptible to environmental stress‐induced apoptosis than cells expressing lower levels of YWHAZ. To further test that idea, we overexpressed YWHAZ in RT4 cells, which endogenously express lower levels of YWHAZ than do other bladder cancer cell lines (Figures 4A,B). Forty‐eight hours after gene transfection, the expression levels of genes involved in endopeptidase‐mediated apoptosis were downregulated as compared to vector‐treated cells, including the DNA break sensor PARP1, pro‐apoptosis BCL‐2 proteins (BAK1 and BAX), and several caspase enzymes (CASP3, CASP7, CASP10) involved in apoptosis initiation and activation (Figure 4C). When RT4 cells were treated with clinical chemotherapy drugs, cells expressing YWHAZ showed higher tolerance/survival activity than cells transfected with empty vector (IC_50_ for doxorubicin: 602.6 versus 79.4 nm; IC_50_ for cisplatin: 20.9 versus 5.2 μm) (Figure 4D). Similarly, YWHAZ overexpression slowed the process of cell death triggered by 6 Gy irradiation in RT4 cells as compared to vector‐treated cells (Figure 4E). Apoptosis analyses using annexin‐V staining also confirmed survival advantages of YWHAZ overexpression when cells were treated with chemotherapy drugs or ionizing radiation (Figure 4F).

### YWHAZ serves as a potential target for treating bladder cancer

**Figure 5:**
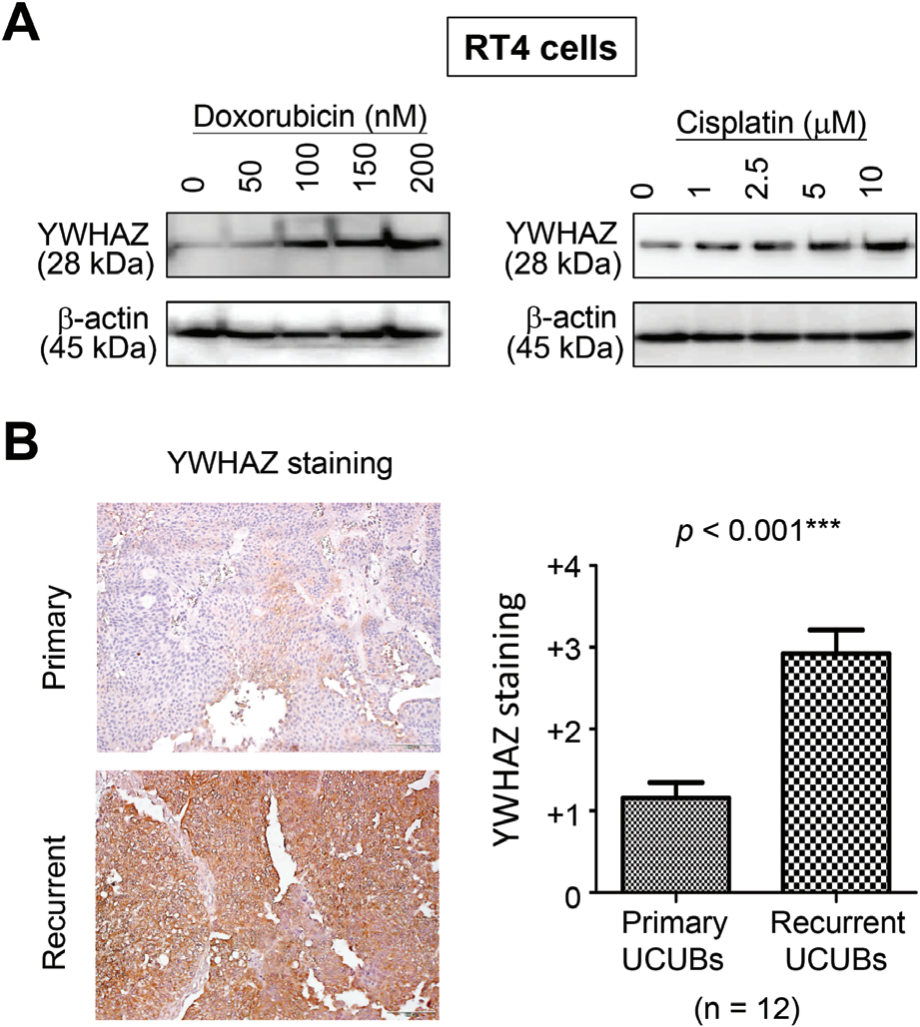
YWHAZ overexpression promotes chemo‐resistance. (A) Western blotting was performed to detect YWHAZ levels in RT4 cells showing drug resistance after exposure to various concentrations of doxorubicin or cisplatin. (B) Representative IHC images of paired UCUBs before (primary) and after (recurrent) chemotherapy (left). YWHAZ staining scores were shown in the bar chart using data from 12 paired UCUBs (right).

Because our data suggest YWHAZ exerts a suppressive effect on apoptosis in bladder cancer, we next endeavored to generate several doxorubicin‐ or cisplatin‐resistant clones from RT4 cells. Notably, exposing the cells to different concentrations of drug induced changes in YWHAZ levels that were well correlated with the cells drug tolerance (Figure 5A). Twelve paired UCUBs before and after chemotherapy were collected for YWHAZ staining, and our data confirmed YWHAZ levels in UCUBs were increased during clonal evolution driven by drug treatments (Figure 5B). These findings suggest that YWHAZ plays an active role in mediating resistance to chemotherapeutic agents.

**Figure 6:**
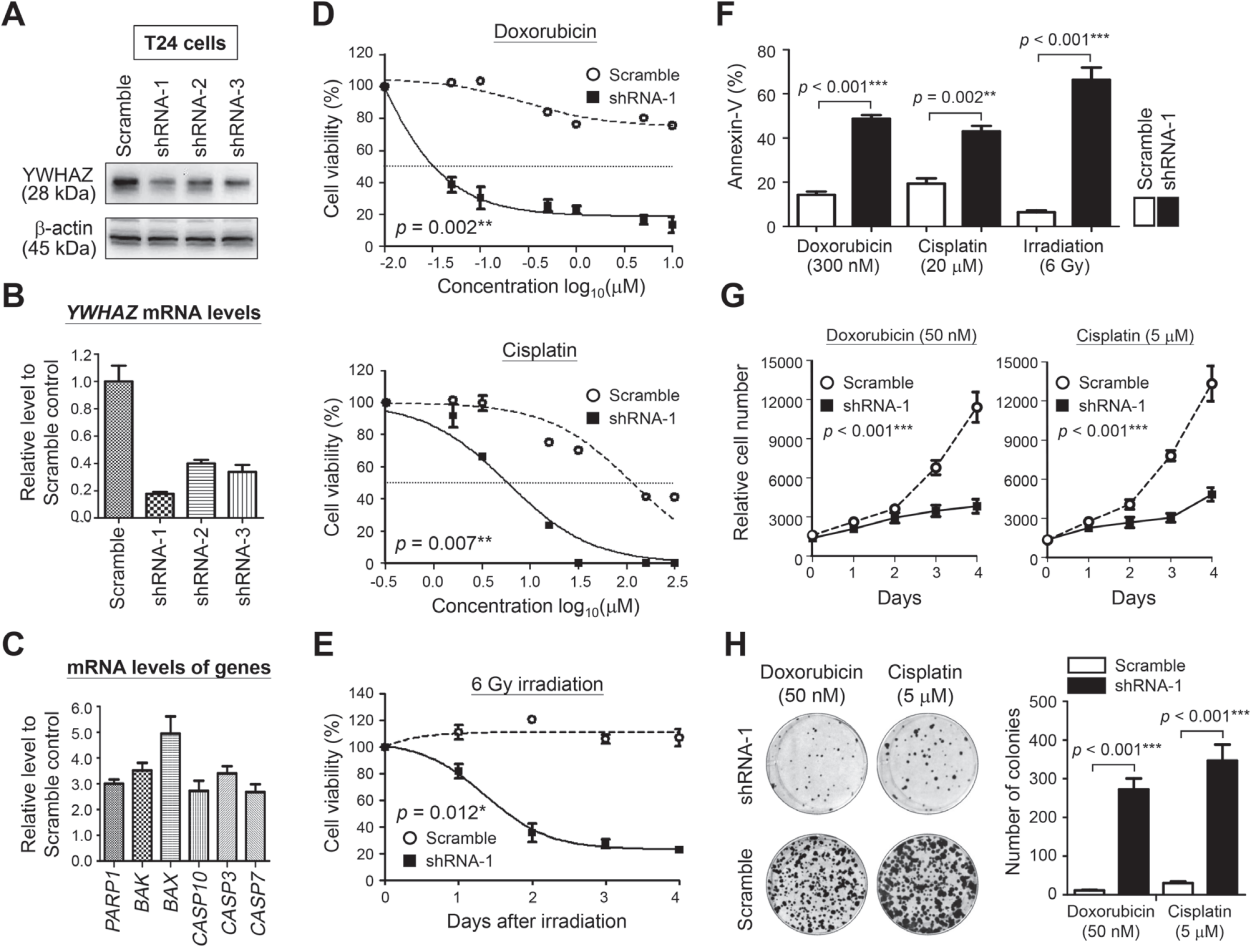
YWHAZ downregulation sensitizes cells to chemo/radiotherapy and suppresses cell growth. (A) Western blotting was performed to detect changes of YWHAZ levels in T24 cells treated with different shRNAs using β‐actin levels as the internal controls. RT‐qPCR was performed to detect mRNA levels of (B) *YWHAZ* in T24 cells treated with shRNAs and (C) the related apoptosis genes in shRNA‐1‐treated T24 cells. Cells treated with ‘scramble’ served as the control in panels A to C. (D) T24 cells transfected with shRNA‐1 were treated with the indicated concentrations of doxorubicin or cisplatin. Cell viability was measured by Alamar Blue assays 4 days after drug treatments. (E) T24 cells transfected with shRNA‐1 were treated with 6 Gy radiation, after which cell viability was assessed every 24 h for 4 days. (F) Incidence rate of preapoptosis among treated cells 48 h after chemo−/radio‐therapy was determined by annexin‐V staining. (G) Proliferation of shRNA‐1‐treated cells was monitored for 4 days under the treatment of 50 nm doxorubicin or 5 μm cisplatin. (H) Colony formation activity was studied on shRNA‐1‐treated T24 cells after 2 weeks treatment with 50 nm doxorubicin or 5 μm cisplatin (left). Colony numbers per 35‐mm dish are shown in bar charts (right). Cells treated with ‘scramble’ served as the controls in panels D to H. Data were expressed as means ± SD from five replicates in each experimental group. Paired *t*‐tests were performed to evaluate differences between ‘scramble’‐ and shRNA‐1‐treated cells. *p < 0.05, **p < 0.01, ***p < 0.001.

To test whether YWHAZ could serve as a potential target to reverse chemo/radioresistance, gene knockdown was performed using specific shRNAs in T24 cells, which were selected because of their relatively high endogenous YWHAZ levels (Figures 4A,B). The shRNA‐1 was chosen in our study because it showed highest gene reduction activity both by western blotting (Figure 6A) and RT‐qPCR (Figure 6B). Gene expression levels of the related pro‐apoptosis genes were found upregulated in T24 cells after *YWHAZ* knockdown (Figure 6C). When treated with chemotherapy drugs, the viability of shRNA‐1‐treated T24 cells was significantly lower than mock‐treated cells (IC_50_ for doxorubicin: 30.9 nm versus more than 10 μm; IC_50_ for cisplatin: 5.6 versus 125.9 μm) (Figure 6D,F). Moreover, reducing YWHAZ levels in T24 cells using shRNA‐1 also dramatically sensitized the cells to 6 Gy irradiation and markedly increased cell death. By contrast, the same treatment had no effect on mock‐treated cells (Figures 6E,F). To further assess the potential therapeutic benefits of inhibiting YWHAZ, we co‐cultured the cells with a low concentration of doxorubicin or cisplatin. When exposed to chemotherapeutic agents, the growth of shRNA‐1‐treated cells was significantly suppressed such that only a few colonies were detected, whereas the mock‐treated cells continued to grow and formed numerous colonies (Figures 6G,H). Our data support the point of view that YWHAZ overexpression is required for UCUB cells to adapt and survive under therapeutic stresses.

## Discussion

In this study, we confirmed *YWHAZ* amplification/overexpression in around 20% of UCUB samples; moreover, this genetic alteration was more frequently detected in MIBC than NMIBC (Figures 1 and 2). In addition, YWHAZ overexpression was found to be a potential prognostic marker indicative of poor clinical outcomes and shorter survival times (Figure 2 and Table 1). Mapping downstream signaling using GSEA and STRING revealed the key role played by YWHAZ in anti‐apoptosis pathways through downregulation of genes involved in caspase‐mediated cell death (Figure 3). A cell‐based functional study further confirmed that YWHAZ overexpression can drive bladder cancer toward resistance to chemo‐/radio‐therapy which can be proven by IHC staining on recurrent UCUBs after chemotherapy (Figures 4 and 5). Conversely, gene knockdown using a specific shRNA in YWHAZ‐overexpressing bladder cancer cells triggered marked increases in cell death after drug or radiation treatments (Figure 6). These data suggest YWHAZ a potent cancer driver that provides survival advantages against environmental stresses to UCUB cells. Consequently, targeting YWHAZ may be an effective strategy to increase the therapeutic efficacy of anticancer therapies.

*YWHAZ* amplification is also reported to be clinically significant in other cancer types, including breast 12, prostate 18, 20, lung 16, and oral 15, 17 cancers. Although YWHAZ has also been reported to act as a tumor suppressor during bladder cancer development 32, 33, our results consistently show that YWHAZ overexpression negatively regulates apoptosis, enabling cellular adaptation to the presence of chemotherapeutic drugs. In contrast to anti‐apoptotic effects, other proteins participating in diverse functions/pathways within cells were found to be upregulated along with YWHAZ overexpression (see supplementary material, Tables S3 and S4). Interestingly, some upregulated effectors implies altered cellular metabolism/homeostasis in cells overexpressing YWHAZ. For example, PYCR1 overexpression can alter amino acid metabolism via the glutamine‐proline regulatory axis 34, which promotes aggressiveness in human cancers 35, 36, 37, and is thus considered to be a useful therapeutic target 37, 38. Similarly, upregulation of SLC2A6 (A.K.A. GLUT6) was reported to promote glucose uptake 39, while POLR2K upregulation was found to increase nucleic acid metabolism 40 in aggressive cancers. Further study is therefore needed to investigate the impact of YWHAZ on bladder cancer metabolism.

In our study, we also found that *YWHAZ* amplification/overexpression associated with downregulation of innate immune responses, suggesting its involvement in tumor microenvironment remodeling (Figure 3A). In recent years, immune checkpoint blockage therapy has shown unprecedented anticancer activity against metastatic UCUBs 41. Nevertheless, more than half of patients using this approach receive no benefit, and some even have a worse outcome 42, 43, 44, indicating the need for a useful biomarker predictive of the therapeutic outcome of such treatments. It is known that innate immune system activation is critical for triggering spontaneous adaptive immune responses against cancer during immunotherapy 45, 46. Thus, combinational therapy to boost immune responses by inducing cytosolic DNA with irradiation or inflammatory tumor death using a bacterial adjuvant (e.g. Bacillus Calmette–Guérin treatment) were proposed as possible strategies to improve the therapeutic efficacy of immunotherapy for UCUB 41, 46, 47. In UCUBs with *YWHAZ* amplification/overexpression, genes involved in interferon signaling, TLR‐4 signaling, inflammasome network, antigen presentation/TCR recognition, and CD28 co‐stimulation were found significantly downregulated (see supplementary material, Table S4). More detailed studies are needed to determine whether YWHAZ could be used as a biomarker to define patients eligible for immune checkpoint blockage therapy and whether anti‐YWHAZ approach could be a useful strategy for improving the therapeutic efficacy of immunotherapy.

Although we defined the tendency to detect *YWHAZ* amplification/overexpression in UCUBs at more advanced stages, we are not yet able to confirm whether YWHAZ functions as a molecular link to drive malignant transition from NMIBC to MIBC. Using a 2D cell culture system, we found limited effects of YWHAZ levels on cell proliferation and invasion, either by protein overexpression in RT4 cells or gene knockdown in T24 cells (see supplementary material, Figure S2). But still, our GSEA analysis indicated the activation of RAP1 signaling pathway in YWHAZ‐overexpressing UCUBs (see supplementary material, Figure S1 and Table S4). Because RAP1 signaling pathway has been proven to be critical in controlling cell migration/invasion and adhesion dynamics via cadherin or integrin signaling 48, 49, 50, an environment that provides more cell–cell contact and cell–matrix adhesion might be necessary to study the impact of YWHAZ on cancer behaviors. In addition, recent studies indicate that YWHAZ acts in concert with the TGF‐β axis to control cancer‐stromal cell interaction and epithelial–mesenchymal transition 21, 26, 51, 52, which suggests other microenvironmental factors are involved in YWHAZ‐mediated carcinogenesis and cancer progression. Organoid cell cultures in a 3D condition or PDX models of tumors overexpressing YWHAZ may provide physiologically relevant contexts for us to better understand the potential roles of YWHAZ in determining UCUB invasiveness.

Since YWHAZ has been implicated in the carcinogenesis of many cancer types, the present study provides a more comprehensive investigation to address its functional roles in UCUB development. In addition to apoptosis regulation and TGF‐β signaling, we also revealed the potential involvement of YWHAZ in regulating critical cancer phenotypes, including cancer metabolism, innate immune responses, and T cell activation. Nevertheless, the association of *YWHAZ* amplification/overexpression to clinical outcomes and other relevant factors was evaluated retrospectively, our findings need to be confirmed with a prospective analysis on another cohort. With diverse cellular functions, YWHAZ could be a useful therapeutic target for treating advanced UCUB, one that enables achievement of better clinical outcomes when used in combination with other therapies.

## Author contributions statement

SCL and JJCS designed the experiments and validated the data. CCY, CFL, IHC, MTL, GK, and CYC collected clinical samples, recorded clinical notes, and performed clinical association study. CFL, IHC, MTL, and CYC prepared the tissue microarray, carried out IHC staining, and then did sample scoring. ZJL, GK, CMC, and TH made the gene constructs, conducted cell‐based studies, and analyzed the data. IHC, PKK and GK performed data mining. CCY, CFL, SCL, and JJC confirmed the data and wrote the manuscript.

**SUPPLEMENTARY MATERIAL ONLINE**

**Figure S1**. Reactomes/pathways associated with *YWHAZ* amplification/overexpression in UCUBs

**Figure S2**. Impacts of YWHAZ expression levels on cell proliferation and invasion in bladder cancer cells

**Table S1**. Sequences of oligonucleotide primers used in this study

**Table S2**. Genes involved in the chromosome 8q22.3 amplicon in urothelial carcinoma of urinary bladder (UCUB)

**Table S3**. Top 20 genes concurrently up‐regulated with *YWHAZ* amplification/overexpression in UCUBs

**Table S4**. Gene set enrichment analyses of genes concurrently up‐ or downregulated with *YWHAZ* amplification/overexpression in UCUBs

## Supporting information


**Figure S1**. Reactomes/pathways associated with *YWHAZ* amplification/overexpression in UCUBs

**Figure S2**. Impacts of YWHAZ expression levels on cell proliferation and invasion in bladder cancer cells

**Table S1** Sequences of oligonucleotide primers used in this study

**Table S2** Genes involved in the chromosome 8q22.3 amplicon in urothelial carcinoma of urinary bladder (UCUB)

**Table S3** Top 20 genes concurrently upregulated with *YWHAZ* amplification/overexpression in UCUBs

**Table S4** Gene set enrichment analyses of genes concurrently up‐ or downregulated with *YWHAZ* amplification/ overexpression in UCUBs
