# Supplementary material for: YWHAZ amplification/overexpression defines aggressive bladder cancer and contributes to chemo‐/radio‐resistance by suppressing caspase‐mediated apoptosis
Source: J Pathol. 2019 Apr 29;248(4):476–87. doi: 10.1002/path.5274 (PMC6767422; doi:10.1002/path.5274)
Supplement: Supplementary file 1 — Figure S1. Reactomes/pathways associated with YWHAZ amplification/overexpression in UCUBs [file PATH-248-476-s001.docx]

***YWHAZ* amplification/overexpression defines aggressive bladder cancer and contributes to chemo-/radio-resistance by suppressing caspase-mediated apoptosis**

Yu C-C *et al*. *J Pathol* DOI: 10.1002/path.5274

**Supplementary Figure S1.** Reactomes/pathways associated with *YWHAZ* amplification/overexpression in UCUBs. Gene set enrichment analysis (GSEA) was performed using mRNA expression data from the TCGA group. In addition to two major pathways (*p* = 0.001) indicated in Figure 3, other potent pathways (*p* < 0.01) were shown as concurrent (A) up-regulated and (B) down-regulated reactomes.
