# Supplementary material for: YWHAZ amplification/overexpression defines aggressive bladder cancer and contributes to chemo‐/radio‐resistance by suppressing caspase‐mediated apoptosis
Source: J Pathol. 2019 Apr 29;248(4):476–87. doi: 10.1002/path.5274 (PMC6767422; doi:10.1002/path.5274)
Supplement: Supplementary file 2 — Figure S2. Impacts of YWHAZ expression levels on cell proliferation and invasion in bladder cancer cells [file PATH-248-476-s002.docx]

***YWHAZ* amplification/overexpression defines aggressive bladder cancer and contributes to chemo-/radio-resistance by suppressing caspase-mediated apoptosis**

Yu C-C *et al*. *J Pathol* DOI: 10.1002/path.5274

**Supplementary Figure S2.** Impacts of YWHAZ expression levels on cell proliferation and invasion in bladder cancer cells. (A) RT4 cells were transfected with YWHAZ expression vector and cells treated with the empty vector were utilized as the control. (B) T24 cells were treated with shRNA-1 to knockdown the endogenous YWHAZ. Cells treated with scramble were used as the control. Cell growth (*upper* panel in both A and B) was measured every day by MTT assay for 4 days. Cell invasiveness (*lower* panel in both A and B) was evaluated in a Transwell system pre-coated with 50 μl Matrigel. Cells penetrated through the gel were counted by Giemsa staining. Data were expressed as means ± SD from five replicates in each experimental group.
