# Supplementary material for: YWHAZ amplification/overexpression defines aggressive bladder cancer and contributes to chemo‐/radio‐resistance by suppressing caspase‐mediated apoptosis
Source: J Pathol. 2019 Apr 29;248(4):476–87. doi: 10.1002/path.5274 (PMC6767422; doi:10.1002/path.5274)
Supplement: Supplementary file 3 — Table S1 Sequences of oligonucleotide primers used in this study [file PATH-248-476-s003.doc]

***YWHAZ* amplification/overexpression defines aggressive bladder cancer and contributes to chemo-/radio-resistance by suppressing caspase-mediated apoptosis**

Yu C-C *et al*. *J Pathol* DOI: 10.1002/path.5274

**Supplementary Table S1. Sequences of oligonucleotide primers used in this study**

| **Gene symbol** | **Reference sequence** | **Forward primer sequence^a,b^** | **Reverse primer sequence^a,b^** |
| --- | --- | --- | --- |
| *YWHAZ* | NM_003406.3 | 5’-CAACAAGCATACCAAGAAG-3’ | 5’-TCATAATAGAACACAGAGAAGT-3’ |
| *PARP1* | NM_001618.3 | 5’-TTCAGCAGATAAGCCATT-3’ | 5’-CTTCACTTCATCCTTGTTC-3’ |
| *BAK* | NM_001188.3 | 5’-GAGAAGGACTATCAACAC-3’ | 5’-AGTTCAAGTATTCACAGTT-3’ |
| *BAX* | NM_138761.3 | 5’-AAGAAGCTGAGCGAGTGT-3’ | 5’-GGCGGCAATCATCCTCTG-3’ |
| *CASP10* | NM_032977.3 | 5’-CAGTTGCCATTGACAGAA-3’ | 5’-TGAAGCCAGTGTTATCCA-3’ |
| *CASP3* | NM_004346.3 | 5’- GACATACTCCTTCCATCAA-3’ | 5’-ATTCATAGCACAGCATCA-3’ |
| *CASP7* | NM_001227.4 | 5’-TTGTATGTCTGTTACCTTGT-3’ | 5’-ACTCTACCACTGTTACCA-3’ |
| *GAPDH* | NM_002046.6 | 5’-CTCTGGTAAAGTGGATATTGT-3’ | 5’-GGTGGAATCATATTGGAACA-3’ |

^a^The SYBR Green-based primers were designed by OligoArchitect^TM^ online (http://www.oligoarchitect.com/SYBRGreenSearchServlet).

^b^QPCR cycles were performed by using an ABI 2720 Thermal Cycler (Applied Biosystems, Carlsbad, CA, USA) and the cycling conditions were: 95 °C for 10 min, followed by 40 cycles of 95 °C for 15 s, 55 °C for 15 s, 72 °C for 15 s, and finally 72 °C for 5 min.
