# Supplementary material for: YWHAZ amplification/overexpression defines aggressive bladder cancer and contributes to chemo‐/radio‐resistance by suppressing caspase‐mediated apoptosis
Source: J Pathol. 2019 Apr 29;248(4):476–87. doi: 10.1002/path.5274 (PMC6767422; doi:10.1002/path.5274)
Supplement: Supplementary file 4 — Table S2 Genes involved in the chromosome 8q22.3 amplicon in urothelial carcinoma of urinary bladder (UCUB) [file PATH-248-476-s004.doc]

***YWHAZ* amplification/overexpression defines aggressive bladder cancer and contributes to chemo-/radio-resistance by suppressing caspase-mediated apoptosis**

Yu C-C *et al*. *J Pathol* DOI: 10.1002/path.5274

**Supplementary Table S2. Genes involved in the chromosome 8q22.3 amplicon in urothelial carcinoma of urinary bladder (UCUB)**

| **Gene symbol** | **Reference sequence** | **Molecular function^a^** | **Gene description** |
| --- | --- | --- | --- |
| *CPQ* | NM_016134 | metallodipeptidase activity | carboxypeptidase Q |
| *MTDH* | NM_001363137 | endoplasmic reticulum membrane; RNA polymerase II transcription factor binding | metadherin |
| *LAPTM4B* | NM_018407 | integral component of membrane; transporter | lysosomal protein transmembrane 4 beta |
| *MATN2* | NM_002380 | extracellular matrix; receptor | matrilin 2 |
| *RPL30* | NM_000989 | structural constituent of ribosome | ribosomal protein L30 |
| *ERICH5* | NM_001170806 | uncharacterized | glutamate rich 5 |
| *RIDA* | NM_005836 | deaminase activity | reactive intermediate imine deaminase A homolog |
| *POP1* | NM_001145861 | ribonuclease P/MRP activity | ribonuclease P/MRP subunit |
| *NIPAL2* | NM_024759 | magnesium ion transmembrane transporter | NIPA like domain containing 2 |
| *KCNS2* | NM_020697 | voltage-gated potassium channel complex | potassium voltage-gated channel modifier subfamily S member 2 |
| *STK3* | NM_001256312 | protein kinase activity | serine/threonine kinase 3 |
| *OSR2* | NM_001286841 | nucleic acid binding; zinc finger transcription factor | odd-skipped related transcription factor 2 |
| *VPS13B* | NM_152564 | protein targeting to vacuole; protein transport; Golgi apparatus | vacuolar protein sorting 13 homolog B |
| *COX6C* | NM_004374 | cytochrome-c oxidase activity | cytochrome c oxidase subunit 6C |
| *RGS22* | NM_001286693 | negative regulation of signal transduction | regulator of G protein signaling 22 |
| *FBXO43* | NM_001029860 | negative regulation of meiotic nuclear division | F-box protein 43 |
| *POLR2K* | NM_005034 | RNA polymerase I/II/III activity | RNA polymerase II subunit K |
| *SPAG1* | NM_172218 | GTP binding; hydrolase activity; single fertilization | sperm associated antigen 1 |
| *RNF19A* | NM_183419 | positive regulation of proteasomal ubiquitin-dependent protein catabolic process | ring finger protein 19A, RBR E3 ubiquitin protein ligase |
| *ANKRD46* | NM_001270379 | integral component of membrane | ankyrin repeat domain 46 |
| *SNX31* | NM_001363720 | phosphatidylinositol binding; intracellular protein transport | sorting nexin 31 |
| *PABPC1* | NM_002568 | poly(U) RNA binding | poly(A) binding protein cytoplasmic 1 |
| ***YWHAZ*** | **NM_145690** | **RNA and protein binding; chaperone** | **tyrosine 3-monooxygenase/tryptophan 5-monooxygenase activation protein zeta** |
| *ZNF706* | NM_016096 | negative regulation of transcription | zinc finger protein 706 |
| *GRHL2* | NM_001330593 | transcription factor | grainyhead like transcription factor 2 |
| *NCALD* | NM_001040625 | protein binding; regulation of systemic arterial blood pressure | neurocalcin delta |
| *RRM2B* | NM_001172478 | deoxyribonucleotide biosynthetic process; reductase | ribonucleotide reductase regulatory TP53 inducible subunit M2B |
| *UBR5* | NM_001282873 | protein polyubiquitination; cellular response to DNA damage stimulus | ubiquitin protein ligase E3 component n-recognin 5 |

^a^Bio-functions were defined by the GO database (http://amigo.geneontology.org) or Panther classification system (http://www.pantherdb.org).
