# Supplementary material for: YWHAZ amplification/overexpression defines aggressive bladder cancer and contributes to chemo‐/radio‐resistance by suppressing caspase‐mediated apoptosis
Source: J Pathol. 2019 Apr 29;248(4):476–87. doi: 10.1002/path.5274 (PMC6767422; doi:10.1002/path.5274)
Supplement: Supplementary file 5 — Table S3 Top 20 genes concurrently upregulated with YWHAZ amplification/overexpression in UCUBs [file PATH-248-476-s005.doc]

***YWHAZ* amplification/overexpression defines aggressive bladder cancer and contributes to chemo-/radio-resistance by suppressing caspase-mediated apoptosis**

Yu C-C *et al*. *J Pathol* DOI: 10.1002/path.5274

**Supplementary Table S3. Top 20 genes concurrently up-regulated with *YWHAZ* amplification/overexpression in UCUBs**

| **Gene symbol^a^** | **Log Ratio (T/N)** | **p-value** | **q-value** | **Gene description** | **Known cellular function^b^** |
| --- | --- | --- | --- | --- | --- |
| PYCR1 | 1.27 | 1.64E-17 | 6.93E-14 | Pyrroline-5-carboxylate reductase 1 | Housekeeping enzyme that catalyzes the last step in proline biosynthesis. Can utilize both NAD and NADP, but has higher affinity for NAD. Involved in the cellular response to oxidative stress. |
| PABPC3 | 1.48 | 3.46E-13 | 6.24E-10 | Poly(A) binding protein, cytoplasmic 3 | Binds the poly(A) tail of mRNA. May be involved in cytoplasmic regulatory processes of mRNA metabolism. |
| TTC7B | 0.74 | 1.72E-10 | 1.98E-07 | Tetratricopeptide repeat domain 7B | Component of a complex required to localize phosphatidylinositol 4-kinase (PI4K) to the plasma membrane via direct interactions with EFR3B and FAM126A. The complex acts as a regulator of phosphatidylinositol 4-phosphate (PtdIns(4)P) synthesis. |
| SLC2A6 | 1.2 | 1.89E-10 | 2.00E-07 | Solute carrier family 2, member 6 | Facilitative glucose transporter; binds cytochalasin B with low affinity. |
| SAPCD2 | 0.92 | 3.33E-10 | 3.01E-07 | Suppressor APC domain containing 2 | N/A |
| STK32C | 0.88 | 5.36E-10 | 4.24E-07 | Serine/threonine kinase 32C | N/A |
| ADAM11 | 1.48 | 1.26E-09 | 8.87E-07 | ADAM metallopeptidase domain 11 | Probable ligand for integrin in the brain. This is a non-catalytic metalloprotease-like protein. |
| GPT2 | 0.88 | 2.49E-09 | 1.65E-06 | Glutamic pyruvate transaminase (alanine aminotransferase) 2 | Catalyzes the reversible transamination between alanine and 2-oxoglutarate to form pyruvate and glutamate. |
| AUNIP | 0.77 | 6.46E-09 | 3.71E-06 | Aurora kinase A and ninein interacting protein | Required for the dynamic movement of AURKA at the centrosomes and spindle apparatus during the cell cycle. |
| MAP6D1 | 0.69 | 7.18E-09 | 3.94E-06 | MAP6 domain containing 1 | May have microtubule-stabilizing activity. |
| TMEM52 | 1.24 | 1.01E-08 | 5.10E-06 | Transmembrane protein 52 | N/A |
| SLC19A1 | 0.6 | 1.95E-08 | 8.50E-06 | Solute carrier family 19 (folate transporter), member 1 | Transporter for the intake of folate. Uptake of folate in human placental choriocarcinoma cells occurs by a novel mechanism called potocytosis which functionally couples three components, namely the folate receptor, the folate transporter, and a V-type H(+)-pump. |
| SLC6A6 | 0.68 | 2.42E-08 | 1.02E-05 | Solute carrier family 6 (neurotransmitter transporter, taurine), member 6; | Sodium-dependent taurine and beta-alanine transporter. Chloride ions are necessary for optimal uptake. |
| SLC29A4 | 1.4 | 3.45E-08 | 1.25E-05 | Solute carrier family 29 (nucleoside transporters), member 4 | Functions as a poly-specific transporter for organic cations and biogenic amines. May play a role in regulating central nervous system homeostasis of monoamine neurotransmitters. |
| CBS | 1.33 | 5.99E-08 | 1.89E-05 | Cystathionine-beta-synthase | Hydrolyase catalyzing the first step of the transsulfuration pathway to form L-cystathionine, the precursor of L-cysteine. Also involved in the production of hydrogen sulfide, a gasotransmitter with signaling and cytoprotective effects on neurons. |
| SH3BP5 | 0.87 | 7.49E-08 | 2.21E-05 | SH3-domain binding protein 5 | Plays a negative regulatory role in BTK-related cytoplasmic signaling in B-cells. May be involved in BCR-induced apoptotic cell death. |
| GINS4 | 0.71 | 7.68E-08 | 2.21E-05 | GINS complex subunit 4 | Plays an essential role in the initiation of DNA replication, and progression of DNA replication forks. GINS complex seems to bind preferentially to single-stranded DNA |
| TMEM97 | 1.21 | 7.69E-08 | 2.21E-05 | Transmembrane protein 97 | Plays a role as a regulator of cellular cholesterol homeostasis. May function as sterol isomerase. |
| DENND2A | 0.96 | 1.45E-07 | 3.47E-05 | DENN/MADD domain containing 2A | May activate RAB9A/9B and promotes the exchange of GDP to GTP, converting inactive GDP-bound Rab proteins into their active GTP- bound form. May play a role in late endosomes back to trans-Golgi network/TGN transport. |
| FGFR4 | 1.28 | 1.62E-07 | 3.80E-05 | Fibroblast growth factor receptor 4 | Acts as cell-surface receptor for fibroblast growth factors and plays a role in the regulation of cell proliferation, differentiation and migration, and in regulation of lipid metabolism, bile acid biosynthesis, glucose uptake, vitamin D metabolism and phosphate homeostasis. |

^a^To reduce possible bias caused by co-amplification in the chromosome 8q22.3 region, we only consider co-upregulated genes from other chromosomes with log Ratios > 0.3, *p*-values < 0.05 and *q*-value < 0.05.

^b^Known gene functions were defined by the STRING database (https://string-db.org/).
