# Supplementary material for: YWHAZ amplification/overexpression defines aggressive bladder cancer and contributes to chemo‐/radio‐resistance by suppressing caspase‐mediated apoptosis
Source: J Pathol. 2019 Apr 29;248(4):476–87. doi: 10.1002/path.5274 (PMC6767422; doi:10.1002/path.5274)
Supplement: Supplementary file 6 — Table S4 Gene set enrichment analyses of genes concurrently up‐ or downregulated with YWHAZ amplification/ overexpression in UCUBs [file PATH-248-476-s006.doc]

***YWHAZ* amplification/overexpression defines aggressive bladder cancer and contributes to chemo-/radio-resistance by suppressing caspase-mediated apoptosis**

Yu C-C *et al*. *J Pathol* DOI: 10.1002/path.5274

**Supplementary Table S4. Gene set enrichment analyses of genes concurrently up- or down-regulated with *YWHAZ* amplification/ overexpression in UCUBs^a^**

| **Reactome / Pathway^b^** | **ES** | **NES** | **NOM *p*-value** | **FDR *q*-value** | **FWER *p*-value** |
| --- | --- | --- | --- | --- | --- |
| **Associated with concurrently up-regulated genes** |  |  |  |  |  |
| RAP1 signaling | 0.613 | 1.822 | 0.007 | 0.432 | 0.488 |
| Packaging of telomere ends | 0.674 | 1.804 | 0.014 | 0.304 | 0.536 |
| Metabolism of vitamins and co-factors | 0.449 | 1.747 | 0.017 | 0.272 | 0.687 |
| RNA Pol III transcription | 0.511 | 1.673 | 0.018 | 0.330 | 0.845 |
| RNA Pol I / Pol II / Pol III and mitochondrial transcription | 0.538 | 1.845 | 0.019 | 0.529 | 0.439 |
| Transcription | 0.468 | 1.722 | 0.021 | 0.272 | 0.749 |
| Deposition of new CENPA containing nucleosomes at the centromere | 0.632 | 1.779 | 0.026 | 0.267 | 0.604 |
| Meiosis | 0.550 | 1.799 | 0.027 | 0.264 | 0.545 |
| Peroxisomal lipid metabolism | 0.624 | 1.731 | 0.029 | 0.279 | 0.728 |
| RNA Pol I transcription | 0.585 | 1.806 | 0.030 | 0.372 | 0.529 |
| Meiotic synapsis | 0.543 | 1.764 | 0.031 | 0.265 | 0.651 |
| **Associated with concurrently down-regulated genes** |  |  |  |  |  |
| Apoptotic cleavage of cellular proteins | -0.547 | -1.845 | 0.001 | 0.540 | 0.371 |
| Innate immune system | -0.436 | -1.841 | 0.001 | 0.423 | 0.383 |
| Platelet sensitization by LDL | -0.639 | -1.812 | 0.005 | 0.431 | 0.458 |
| Interferon gamma signaling | -0.701 | -1.961 | 0.007 | 0.486 | 0.132 |
| PI3K events in ErbB4 signaling | -0.497 | -1.775 | 0.007 | 0.364 | 0.56 |
| Activated TLR4 signaling | -0.435 | -1.710 | 0.009 | 0.354 | 0.731 |
| PI3K_AKT activation | -0.456 | -1.665 | 0.009 | 0.405 | 0.826 |
| Interferon gamma signaling | -0.528 | -1.890 | 0.011 | 0.508 | 0.256 |
| Co-stimulation by the CD28 family | -0.572 | -1.797 | 0.013 | 0.406 | 0.498 |
| Generation of second messenger molecules | -0.769 | -1.779 | 0.014 | 0.404 | 0.554 |
| RIG1_MDA5-mediated induction of IFN alpha / beta pathways | -0.471 | -1.760 | 0.014 | 0.366 | 0.609 |
| PI3K events in ErbB2 signaling | -0.461 | -1.665 | 0.016 | 0.383 | 0.827 |
| Inflammasomes | -0.663 | -1.714 | 0.018 | 0.369 | 0.724 |
| GPVI-mediated activation cascade | -0.549 | -1.655 | 0.020 | 0.369 | 0.842 |
| Glycolysis | -0.603 | -1.725 | 0.023 | 0.367 | 0.692 |
| Cytokine signaling in immune system | -0.431 | -1.697 | 0.026 | 0.362 | 0.755 |
| Nuclear events kinase and transcription factor activation | -0.581 | -1.692 | 0.026 | 0.351 | 0.767 |
| TCR signaling | -0.579 | -1.72 | 0.031 | 0.399 | 0.692 |
| Antigen presentation folding assembly and peptide loading of Class-I MHC | -0.671 | -1.662 | 0.041 | 0.371 | 0.831 |
| Interferon alpha / beta signaling | -0.595 | -1.734 | 0.043 | 0.408 | 0.673 |

^a^Abbreviations: ES, enrichment score; NES, normalized enrichment score; NOM, nominal; FDR, false discovery rate; FEWR, familywise-error rate.

^b^Reactomes or pathways with NOM *p*-values < 0.05, FDR *q*-values < 0.60 and FEWR *p*-values < 0.85 were considered as significantly relevant with *YWHAZ* amplification/overexpression.
